# Major dietary patterns and dietary inflammatory index in relation to dyslipidemia using cross-sectional results from the RaNCD cohort study

**DOI:** 10.1038/s41598-023-46447-8

**Published:** 2023-11-04

**Authors:** Yahya Pasdar, Fardin Moradi, Sahar Cheshmeh, Mohammad Sedighi, Amir Saber, Shima Moradi, Mitra Bonyani, Farid Najafi

**Affiliations:** 1 Department of Nutrition Sciences, Research Center for Environmental Determinants of Health (RCEDH), Health Institute, Kermanshah University of Medical Sciences, Kermanshah, Iran; 2 Student Research Committee, School of Nutritional Sciences and Food Technology, Kermanshah University of Medical Sciences, Kermanshah, Iran; 3 Molecular and Experimental Nutritional Medicine Department, University of Potsdam, Nuthetal, Germany; 4 Medical Education Development Center, Kermanshah University of Medical Sciences, Kermanshah, Iran; 5 Research Center for Environmental Determinants of Health (RCEDH), Kermanshah University of Medical Sciences, Kermanshah, Iran

**Keywords:** Dyslipidaemias, Nutrition disorders

## Abstract

Dyslipidemia can increase the risk of heart attack and stroke due to the restriction of blood flow through the blood vessels. Dietary modification is an appropriate approach to reducing this phenomenon. This cross-sectional study aimed to evaluate major dietary patterns and the dietary inflammatory index (DII) in relation to dyslipidemia. 5954 participants in the Ravansar non-communicable diseases (RaNCD) cohort study were eligible for this study. Dyslipidemia was diagnosed based on the lipid profile under consideration of the RaNCD physician. Dietary patterns were assessed by principal component analysis. The three identified dietary patterns included (1) plant-based pattern; (2) high protein and sugar pattern; and (3) energy-dense dense pattern. DII was also calculated based on the dietary information from a validated semi-quantitative food frequency questionnaire (FFQ). We found that higher adherence to DII was significantly associated with increased odds of dyslipidemia after adjusting for age, sex, and physical activity (OR: 1.24; CI 95% 1.09–1.42). Additionally, higher adherence to the high protein and sugar diet and an energy-dense diet was significantly associated with higher odds for dyslipidemia (OR: 1.31; CI 95% 1.16–1.49) and (OR: 1.28; CI 95% 1.12–1.46). Nevertheless, according to our results, following plant-based diet had no association with dyslipidemia in both crude and adjusted models. Our findings revealed that greater adherence to DII, a high-protein, high-sugar diet, and an energy-dense diet can have undesirable effects on dyslipidemia.

## Introduction

Dyslipidemia is an important predisposing factor in increasing the risk of cardiovascular diseases (CVDs)^1,2^. Studies have shown that dyslipidemia is associated with a lipid profile imbalance, including higher levels of triglycerides (TG), low-density lipoprotein cholesterol (LDL-C), and total cholesterol (TC) while the amount of high-density lipoprotein cholesterol (HDL-C) is low^3^. In the last 30 years on the Asian continent, the amount of mortality from CVDs has increased from 5.6 million to 10.8 million^4^. Dyslipidemia causes about 4 million deaths from CVDs worldwide^5^.

The main contributors to dyslipidemia are an inactive lifestyle, genetic factors, hormonal abnormalities, obesity, and diet^6,7^. In recent years, the dietary pattern approach has been the focus of many researches and has been widely used in examining the international effects of nutrients together^8^. In fact, the dietary pattern approach is related to the amounts, proportions, variety, or combination of different foods, drinks, and nutrients in the usual diet and their consumption frequency^9^. Examining dietary patterns conceptually provides a more realistic picture of the overall diet^8^. Therefore, due to the interaction of nutrients in the consumed meal, it is not possible to separate the components of the food, and for this reason, the use of these indicators and food patterns has been expanded as a way to evaluate the effects of the overall dietary intake^10^.

The dietary inflammatory index (DII) was developed to estimate the inflammatory potential.

of foodandwas first introduced by Shivappa et al.^11^. The DII has been used in many epidemiological studies involving different ethnicities and different health outcomes^12,13^ where higher adherence to DII was related to an increase pro-inflammatory cytokines such as c- reactive protein (CRP), interleukin 6 (IL-6), and tumor necrosis factor- a (TNF-a)^14^. DII can be used to evaluate the inflammatory status of diets, including anti-inflammatory or pro-inflammatory properties^11^.

According to the high prevalence of dyslipidemia in the Ravansar non-communicable diseases (RaNCD) cohort study^15^, the present study was designed with the aim of investigating dietary patterns and DII in relation to dyslipidemia in people.

## Material and methods

### Study design

We designed a cross-sectional study using data from the RaNCD cohort study, a population-based study on the Kurdish population (4764 men and 5283 women) aged 35–65 years, Ravansar, Kermanshah province, Western Iran. The RaNCD cohort study, subset of the PERSIAN (Prospective Epidemiological Research Studies in Iran) mega cohort study, was approved by the Ethics Committees in the Ministry of Health and Medical Education, the Digestive Diseases Research Institute, Tehran University of Medical Sciences, Iran^16,17^. All procedures for the RaNCD cohort study were approved by the Ethics Committee of Kermanshah University of Medical Sciences (ethics approval number: IR.KUMS.REC.1401.507). Furthermore, all participants provided written informed consent.

### Participants

In this study, we did not include pregnant women (n = 138). We further did not include participants who had been diagnosed with CVDs (n = 1684), diabetes (n = 490), hypertension (n = 372), thyroid disease (n = 507), or cancer (n = 57). After that, we excluded participants with unusual energy intake (< 800 kcal/day or ≥ 4200 kcal/day for men and (< 600 kcal/day or ≥ 3500 kcal/day for women) (n = 788) and insufficient data (n = 57). A total of 5954 participants were finally selected for the analysis.

### Physical activity assessment

Physical activity was assessed by the standard questionnaire designed by the PERSIAN mega cohort study, with twenty two questions about the amount of daily activities. The report of this questionnaire was evaluated based on the metabolic equivalent of tasks per hour per day (MET/h/day). Details of this questionnaire was published in the previous study^17^.

### Socioeconomic status (SES)

Participants' SES was measured based on the asset-based approach. Data on household income, housing conditions (e.g. type of home ownership and number of rooms), infrastructure facilities (sanitary facilities, drinking water supply), ownership of a range of durable assets (e.g. car, dishwasher, television, etc.), and level of education (illiterate, under-diploma, diploma, and university) were used to measure individuals' SES. SES was constructed using the principal component analysis (PCA) technique. PCA generates weights for each selected asset and then estimates a continuous index based on the sum of the weights of the variables included in the PCA for each individual. This index was used to categorize individuals into three SES tertiles (from poorest to richest).

### Anthropometric measurement/biochemical assessment

Height and body weight were assessed in the recruitment phase of the RaNCD cohort study. Body mass index (BMI) was calculated by dividing weight (kg) by the square of height (m^2^). Furthermore, waist circumference (WC) was measured by non-stretched and flexible tape in a standing position at the level of the iliac crest by a trained nutritionist. In the RaNCD cohort study, fasting blood samples were obtained from all RaNCD participants and all serum samples were analyzed at the RaNCD laboratory. To assess lipid profile, Concentrations of total cholesterol (TC), high-density lipoproteins (HDL), triglyceride (TG) and low-density lipoproteins (LDL) were analyzed using enzymatic kits (Pars Azmun, Iran)^18^.

### Dyslipidemia

Dyslipidemia was considered to have an LDL cholesterol of ≥ 160 mg/dl and/or total cholesterol of ≥ 240 mg/dl and/or HDL cholesterol of < 40 mg/dl and/or triglycerides of ≥ 200 mg/dl and/or a history of taking medications for dyslipidemia^15^.

### Dietary intake assessment

The dietary intake of RaNCD participants was analyzed using a validated semi-quantitative food frequency questionnaire (FFQ)^19,20^.

### Dietary inflammatory index

Dietary intake information was also used to calculate DII using the Shivapa method^11^. Shivapa et al.^11^ reported that 45 nutrients were associated with one or more inflammatory events, including interleukin-1b (IL-1b), interleukin-6 (IL-6), tumor necrosis factor-a (TNF-a), reactive protein C (CRP), or anti-inflammatory markers, including interleukin-4 (IL-4) and interleukin-10 (IL-10). Therefore, each food parameter that had inflammatory potential was scored + 1. A score of -1 was given to foods that caused a decrease in inflammation or an increase in anti-inflammatory markers, and zero meant no effect on reducing or increasing inflammation.

In this study, based on the RaNCD FFQ questionnaire, 31 food parameters from 45 food items were available to calculate DII, including: vitamin A, vitamin B6, vitamin B12, vitamin C, vitamin D, vitamin E, folic acid, niacin, iron, zinc , selenium, magnesium, beta-carotene, caffeine, thiamin, riboflavin, onion, garlic, tea, omega 3, omega 6, trans fat, saturated fats (SFAs), cholesterol, monounsaturated fatty acids (MUFA), polyunsaturated fatty acids (PUFA), fiber, protein, total fat, carbohydrate, and energy. First, to calculate DII, the intake of each of the mentioned food parameters is subtracted from the global average intake of that food parameter and divided by its global standard deviation, the Z score is obtained, which is standardized for the parameter related to the food itself, and to minimize the effect of right skewness, this value is converted into a percentile score, and the percentile score is multiplied by two and subtracted from the number one. The number obtained in the inflammatory score corresponding to that food parameter is multiplied and the numbers obtained from each food parameter for each person are added together to obtain the total inflammatory score for each person. Finally, all three known food patterns and DII were divided into three groups to check the relationship between different variables. The first tertile and the third tertile represent the least and the most adherence to the respective patterns, respectively.

### Statistical analysis

Quantitative variables were described by mean ± standard deviation (SD), and qualitative variables were reported using frequency (%). The comparison of participants’ baseline characteristics was evaluated using the Chi-square and ANOVA tests based on the tertiles of DII. This FFQ covered 118 food items commonly found in the Kurdish eating pattern. The data from these food items were categorized into 31 food groups (principal components) based on the nutrient content similarity^21^ (Table 1). These 31 food groups were included in PCA as items (input variables). We extracted the major dietary patterns by principal component analysis (PCA) and for factor rotation, we used the varimax rotation method to reduce the number of variables. We identified three main dietary patterns with eigenvalues greater than 1.5 based on the Scree plot and the interpretability of the factors. These dietary patterns were named on the basis of characteristics of the food groups they included (with factor loadings > 0.2). The KMO and Bartlett’s test was 0.798. The radar graph was drawn to better show the factor loadings of food groups in three major dietary patterns. Binary logistic regression in crude and adjusted odds ratios (OR) and 95% confidence intervals (CI) was used to determine the association between dyslipidemia and tertiles of DII and three major dietary patterns. In the adjusted model, we controlled age (continuous), sex (categorical), SES (categorical), and physical activity (continuous) as potential confounders. In all analyses, the first tertile of DII and three major dietary patterns were considered the reference categories. In addition, to better illustrate this association, we considered the figure of linear regression OR across increased DII and three major dietary patterns with adjustment for the mentioned variables in logistic regression. All statistical analyses were conducted using SPSS 20 (IBM Corp, Chicago, IL, USA) and Stata, version 14 (Stata Corp, College Station, TX). P-values were considered significant at the level of < 0.05.

**Table 1:** Food groupings used in the dietary pattern analyses.

Food groups	Dietary components
Leafy vegetables	Cauliflower, lettuce, cucumber, onion, green bean, mushroom, pepper, garlic, turnip, others
Fresh fruits	Melon, watermelon, honeydew melon, plums, prunes, apples, cherries, sour cherries, peaches, nectarine, pear, fig, date, grapes, kiwi, pomegranate, strawberry, banana, persimmon, berry, pineapple, oranges, others
Dried fruits	Dried apricots, Dried berries, raisins, and other type dried fruits
Dairy	Milk, yogurt, yogurt drink (doogh), cheese, chocolate milk, crud
Tomato	Tomato
Carotene-rich vegetables	Yellow squash, carrot
Condiments	Spices, pepper powder, tomato paste, mayonnaise
Pickles	Pickles
Legumes	All type beans, peas, lentils, mung bean, soy
Whole grain	Dark breads (Iranian), wheat, barley
Starchy vegetables	Corn, eggplant, green peas, green squash
Vegetable oil	Vegetable oil
Natural juices	All fruit juices
Butter	Butter, margarine
Olive	Olive and olive oil
Organ meat	Heart, kidney, liver, tongue, brain, offal
Read meat	Beef, lamb, minced meat
Fish	All fish types
Processed meat	Hamburger, sausage, delicatessen meat, pizza
Soft drink	Soft drink
Nuts	Almond, peanut, walnut, pistachio, hazelnut, seeds
Egg	Egg
Poultry	Chicken
Snack	Corn puffs, potato chips, French fries
Sweets and desserts	Cookies, cakes, biscuit, muffins, pies, chocolates, ice, honey, jam, sugar cubes, sugar, candies, others
Tea and coffee	Tea and coffee
Hydrogenated fat	Hydrogenated fats, animal fats including ghee and tallow
Salt	Salt
Potato	Potato
Refined grain	White breads (lavash, baguettes), noodles, pasta, rice

### Ethics approval and consent to participate

All procedures performed in studies involving human participants were in accordance with the ethical standards of the institutional and/or national research committee and with the 1964 Helsinki declaration and its later amendments or comparable ethical standards. This study was approved by the Ethics Committee of Kermanshah University of Medical Sciences (ethics approval number: IR.KUMS.REC.1401.507).

### Informed consent

Written informed consent was obtained from each studied subject after explaining the purpose of the study. The right of the subjects to withdraw from the study at any time and the subject’s information are reserved and will not be published.

## Results

This current study was conducted on data from 5954 RaNCD participants (51.9% men). 40.2% of these participants had dyslipidemia. The mean of weight and BMI significantly increased with higher adherence to DII (P < 0.001). The prevalence of dyslipidemia in the third was significantly higher than in other tertiles of DII (P < 0.001). Table 2 describes the baseline characteristics of the studied participants in total and based on tertiles of dietary inflammatory index.

**Table 2:** Baseline characteristics of studied participants based on tertiles of dietary inflammatory index.

Variables	Total (n = 5954)	Tertiles of dietary inflammatory index			P
		T1 (n = 1984)	T2 (n = 1985)	T3 (n = 1985)	
Age (year)	45.80 ± 7.82*	46.74 ± 8.09	45.58 ± 7.78	45.07 ± 7.50	< 0.001
Sex, male%	51.9	45.4	50.5	59.7	< 0.001
SES					
Low	32.4	45	29	23.4	< 0.001
Moderate	32.5	28.3	34.5	34.9	
High	35	26.7	36.6	41.7	
Weight (kg)	71.87 ± 13.40	69.50 ± 13.11	72.00 ± 13.27	74.12 ± 13.41	< 0.001
BMI (kg/m^2^)	23.78 ± 9.26	23.12 ± 9.01	24.01 ± 9.19	24.22 ± 9.53	< 0.001
WC (cm)	96.05 ± 10.27	95.93 ± 10.41	96.19 ± 10.16	96.02 ± 10.25	0.716
PA (MET/day)	41.28 ± 8.39	40.96 ± 7.32	41.00 ± 8.19	41.87 ± 9.47	0.001
TG (mg/dl)	130.85 ± 74.72	129.30 ± 76.06	128.55 ± 72.34	134.71 ± 75.58	0.018
TC (mg/dl)	183.86 ± 36.54	185.06 ± 37.44	183.43 ± 35.71	183.08 ± 36.43	0.189
HDL (mg/dl)	46.61 ± 11.37	47.93 ± 11.48	46.82 ± 11.53	45.09 ± 10.90	< 0.001
Dyslipidemia,%	40.2	37.1	39.1	44.3	< 0.001
Taking medications, %	1.2	0.6	1.3	1.8	0.278
high LDL levels, %	24.1	24	23.4	23.9	0.511
high TC levels, %	29.3	28.9	29	30.7	0.233
low HDL levels, %	47.4	45.1	47.6	49.7	0.014
Smoking, %	12.8	12.5	13.2	12.6	0.756

*BMI* body mass index, *WC* waist circumference, *PA* physical activity, *TG* triglyceride, *TC* total cholesterol, *HDL* high density lipoprotein, *SES* socioeconomic status.

P-values were obtained ANOVA and Chi square test.

*Mean ± SD.

**Figure 1:**
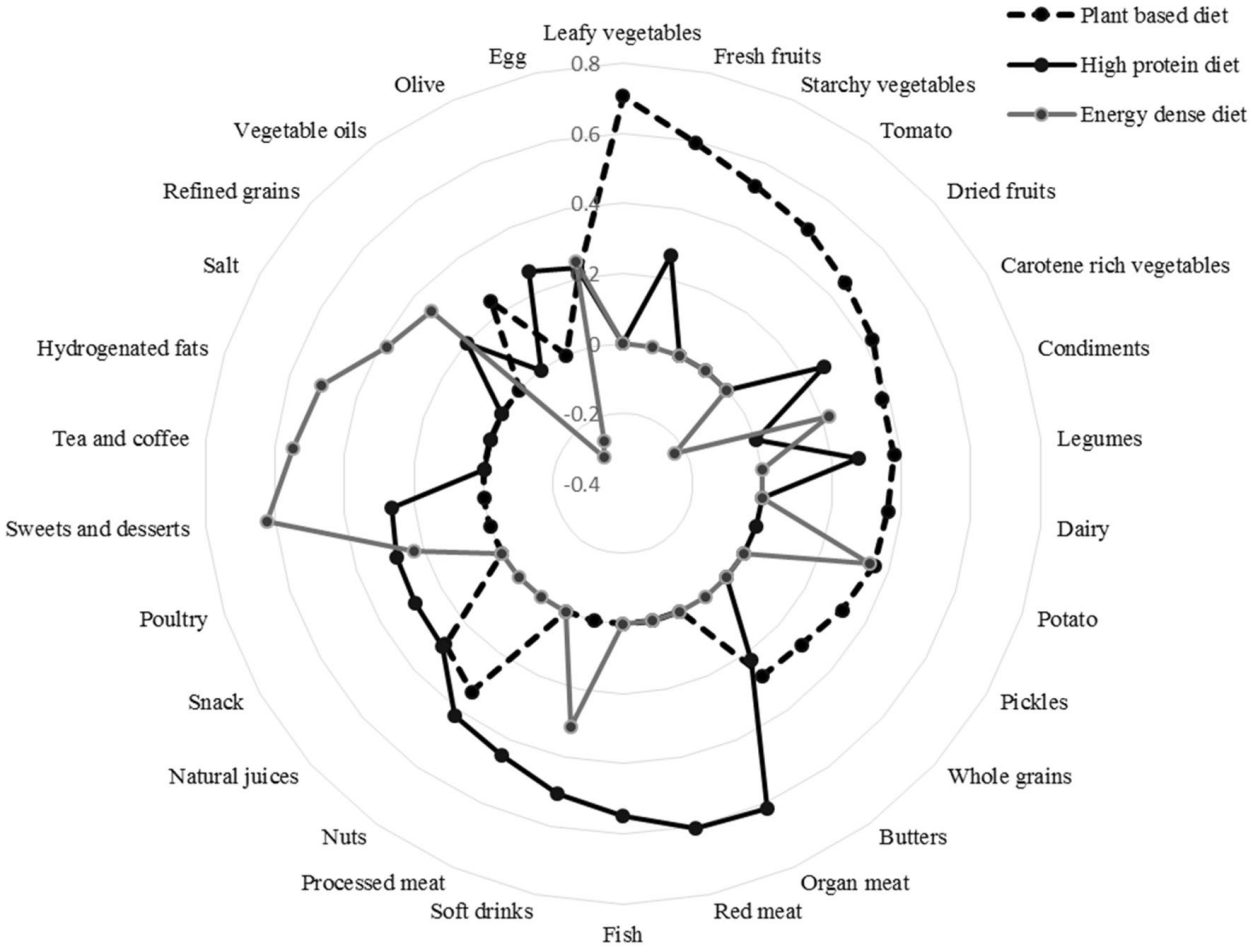
Radar graph for factor loading of food groups in all dietary patterns.

In this present study, three dietary patterns were identified including (1) plant-based diet which was associated with major intake to leafy vegetables, carotene rich vegetables, fresh and dried fruits, starchy vegetables, tomato, potato, condiments, legumes, dairy, pickles, nuts, natural juice, whole grain, vegetable oil, and egg; (2) high protein and sugar diet tend to organ meat, red meat, processed meat, fish, nuts, poultry, sweet and dessert, soft drink, snack, butter, legumes, carotene rich vegetables, refined grain, olive, and, egg; and (3) energy dense diet had the most factor loadings for sweet and dessert, soft drink, tea and coffee, hydrogenated fats, salt, refined grain, potato, egg, condiments, poultry, and negative factor loadings for carotene rich vegetables, vegetable oil, and olive. Factor loadings for all food groups are presented in Table 3 and Fig. 1.

**Table 3:** Factor loading of food groups in all dietary patterns.

Food groups	Major identified dietary pattern		
	Plant based diet	High protein and sugar diet	Energy dense diet
Leafy vegetables	0.707	–	–
Fresh fruits	0.595	0.266	–
Starchy vegetables	0.531	–	–
Tomato	0.498	–	–
Dried fruits	0.455	–	–
Carotene rich vegetables	0.425	0.264	− 0.231
Condiments	0.380	–	0.220
Legumes	0.379	0.278	–
Dairy	0.362	–	–
Potato	0.356	–	0.341
Pickles	0.324	–	–
Whole grains	0.288	–	–
Butters	0.278	0.222	–
Organ meat	–	0.616	–
Red meat	–	0.606	–
Fish	–	0.548	–
Soft drinks	–	0.505	0.309
Processed meat	–	0.451	–
Nuts	0.335	0.420	–
Natural juices	0.285	0.293	–
Snack	–	0.285	–
Poultry	–	0.280	0.230
Sweets and desserts	–	0.265	0.623
Tea and coffee	–	–	0.549
Hydrogenated fats	–	–	0.507
Salt	–	–	0.378
Refined grains	–	0.201	0.337
Vegetable oils	0.243	–	− 0.306
Olive	–	0.261	− 0.269
Egg	0.212	0.229	0.246
Variance, %	13.8	6.52	5.03

Values < 0.2 have been removed for clarity.

**Figure 2:**
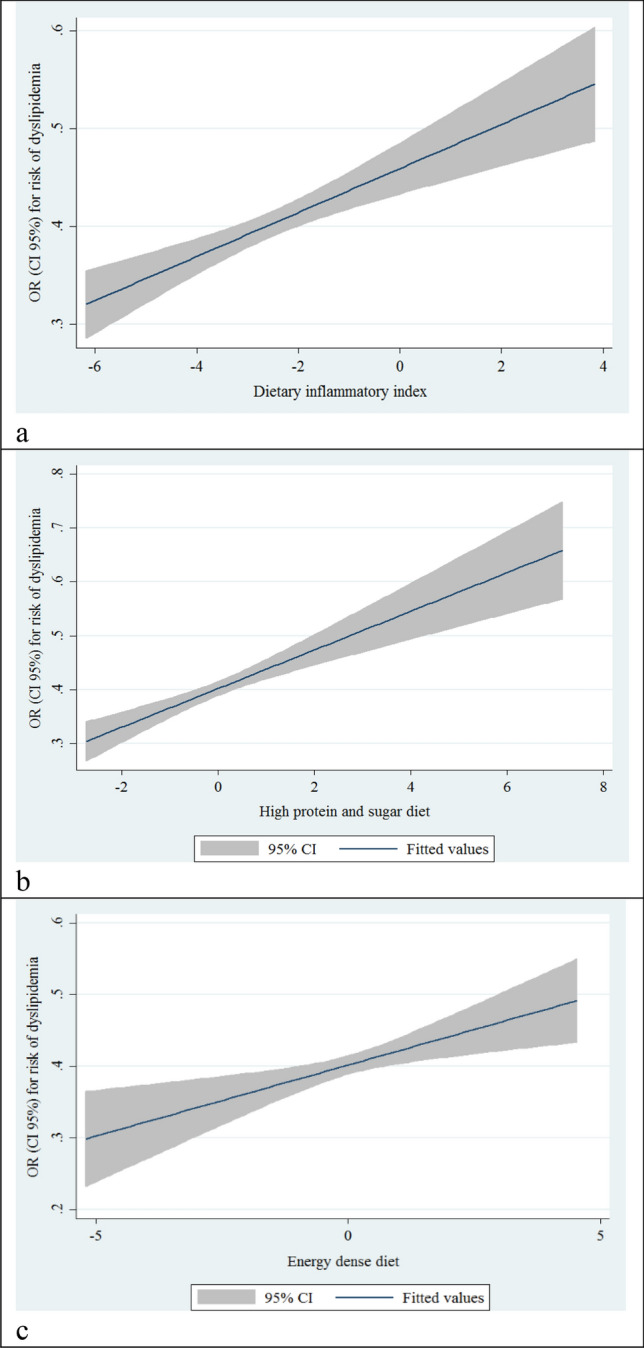
Liner regression odds ratios and 95% confidence intervals for dyslipidemia across categories of (**a**) dietary inflammatory index; (**b**) high protein and sugar diet; and (**c**) energy dense diet.

In this study, binary logistic regression for dyslipidemia across tertiles of DII revealed that greater adherence to DII was associated with significantly increased odds of dyslipidemia (OR: 1.35; CI 95% 1.19–1.53). This association remained after adjusting for age, sex, and physical activity (OR: 1.24; CI 95% 1.09–1.42). (Table 4, Fig. 2a).

**Table 4:** Multivariable-adjusted odds ratios and 95% confidence intervals for dyslipidemia across categories of dietary inflammatory index and major identified dietary patterns.

Dietary patterns	Tertiles of dietary inflammatory index			P- trend
	T1	T2	T3	
DII				
Crude	1	1.09 (0.96–1.24)a	1.35 (1.19–1.53)	< 0.001
Model 1*	1	1.06 (0.93–1.21)	1.24 (1.09–1.42)	0.001
Plant based diet				
Crude	1	0.98 (0.86–1.11)	1.12 (0.99–1.27)	0.072
Model 1**	1	0.99 (0.86–1.13)	1.12 (0.97–1.30)	0.124
High protein and sugar diet				
Crude	1	1.07 (0.94–1.22)	1.31 (1.16–1.49)	< 0.001
Model 1**	1	1.02 (0.9–1.16)	1.18 (1.04–1.37)	0.013
Energy dense diet				
Crude	1	1.04 (0.91–1.18)	1.19 (1.05–1.35)	0.008
Model 1**	1	1.01 (0.97–1.25)	1.28 (1.12–1.46)	< 0.001

*Model 1 adjusted for age, sex, SES, and physical activity.

**Model 1 adjusted for age, sex, SES, physical activity, and energy intake.

^a^OR (CI 95%).

On the other hand, our findings on the relationship between major identified dietary patterns and dyslipidemia showed that higher adherence to the high protein and sugar diet and energy dense were significantly associated with higher odds for dyslipidemia in both crude and adjusted models (OR: 1.31; CI 95% 1.16–1.49) and (OR: 1.28; CI 95% 1.12–1.46). (Table 4, Fig. 2b,c) Nevertheless, according to our results, plant-based diet had no association with dyslipidemia. We did not find any associations adjusted for the mentioned potential confounders. (Table 4).

## Discussion

Findings from our cross-sectional study support the hypothesis of a relationship between a pro-inflammatory diet (as estimated by the DII) and increased dyslipidemia in a Kurdish population.

We observed that greater adherence to DII, high protein and sugar diet or a diet of increased energy intake was associated with higher odds for dyslipidemia.

In our study, it was observed that increased DII was associated with higher serum levels of TG and lower levels of HDL. These findings are in line with Neufcourt et al.,^22^ study and another study in an Iranian population^23^. In addition, it was observed that adherence to an anti-inflammatory dietary pattern that is rich in antioxidants ameliorates the lipid profile^24,25^. Evidence supports an inverse relationship between DII and serum HDL^26^. However, in several studies, there was no association between DII and TG, which may be due to the smaller number of dietary factors used for the calculation of DII^27–29^. Also, similar results are found in relation to HDL^27,28^. The relationship between the anti-inflammatory diet, which contains rich resources of β-Carotene, vitamin C, omega-3, and fiber, and pro-inflammatory diet, such as the western food pattern (rich in energy, hydrogenated fat, and processed foods), has been shown to reduce and increase inflammatory factors such as CRP and IL-6, respectively^30^. According to the evidence, to explain how diets with a higher DII lead to dyslipidemia, it should be said that these foods activate the NF-kB pathway^31^ and can stimulate TG production in the liver and then release it into the blood^32,33^, as well as alters the balance of the lipid profile^34^.

In our study, adherence to high protein and sugar diet was related to increasing odds of dyslipidemia. In terms of effect on cardiovascular risk factors and metabolic syndrome, it is similar to the effect of a Westernized or energy dense dietary pattern^35,36^. A western dietary pattern often has more fast food, fats, and meat and less vegetables and grains^37^. In Zhang et al.'s study, more adherence to this food pattern was associated with an increase in LDL cholesterol^38^. Red and processed meat in the high protein and sugar diet have higher amounts of heme iron and saturated fatty acids, which decrease LDL receptor-mediated clearance^39^. Also, heme iron and saturated fatty acids lead to increased oxidative stress, which is a risk factor for dyslipidemia^39,40^. In other studies, it was observed that if SFAs are replaced with unsaturated fatty acids (UFAs), the number of LDL receptors increases^41,42^.

In our study, the energy dense diet was related to increasing odds of dyslipidemia. This pattern is characterized by high consumption of sweets and refined grains, as well as salt, and low intake of fruits and vegetables. Similar observations were seen in previous studies, including one study that found a significant relationship between the consumption of carbonated drinks and an increased risk of dyslipidemia^43^. In the study of Al-Lahou et al., it was observed that a major intake to refined grains led to a higher risk of dyslipidemia^44^.

According to our findings, the plant based dietary pattern had no association with dyslipidemia, unlike the two patterns mentioned above. Despite this, a recent meta-analysis published in including 30 clinical trials concluded that compared to people eating an omnivorous diet, those who were following a plant-based diet experienced an average reduction in total cholesterol levels of 7% from levels measured at the start of the studies, a 10% reduction in LDL cholesterol levels and a 14% reduction in Apo B levels^45^. It was also stated that plant-based diets have the potential to reduce the atherosclerotic burden caused by atherogenic lipoproteins and thus reduce the risk of cardiovascular diseases^45^. Foods of this pattern are rich sources of fiber, vitamins, and polyphenols similar to the Mediterranean diet and the dietary approaches to stop hypertension (DASH) diet^46,47^, which may have antioxidant and anti-inflammatory properties and may affect the lipid profile^6^. In past studies, it was observed that greater adherence a plant-based dietary pattern was associated with a reduction in TC and LDL^48^ as well as an incidence of dyslipidemia^44^. In a randomized controlled trial, adherence to the DASH diet, compared with the control diet, resulted in a significant decrease in serum triglycerides, and very-low-density lipoprotein cholesterol levels^49^. In a recent study by Antoniazzi et al., it was shown that higher adherence to a Mediterranean diet was associated with better dyslipidemia and low-grade inflammation profiles in familial hypercholesterolemia^50^. The neutral effect of the plant-based diet seems to be because this pattern contains a high factor loading for fructose sources (fruit, juice, and dried fruit), as well as butter especially margarine. The most important metabolic side effects of high fructose consumption are postprandial hypertriglyceridemia, which leads to the activation of protein kinase C, hepatic triglyceride accumulation, and hepatic insulin resistance by increasing visceral fat deposition^51^. Additionally, butter rich in cholesterol, saturated fatty acids, especially trans fatty acids, which increases plasma cholesterol and HDL cholesterol concentration. Hence, the ratio of total cholesterol to HDL remains largely unchanged^52^. Studies have also observed weak or neutral associations between butter consumption and mortality, CVD, diabetes and cancer^53,54^. On the other hand, margarine is a type of butter that is produced from vegetable oils in the hydrogenation process, but it has a higher trans fatty acid content than butter^55^. Excess intake of trans fatty acids can effectively increase LDL levels and subsequently increase the risk of CVDs^56^. As a result, the reason for not observing the relationship between plant-based dietary patterns and dyslipidemia may be that the benefits of fiber, vitamins, and polyphenols have been neutralized by the higher amount of margarine and fructose consumption.

Among the important limitations of this study is its cross-sectional nature, which cannot show a causal relationship between DII and dyslipidemia. Other limitations of this study are the use of an FFQ and the lack of all the food factors that are used in the calculation of the DII. Further well design follow- up studies in this field seem necessary.

In summary, our findings highlighted that higher adherence to energy dense, and high protein and sugary dietary patterns and also a higher DII score can have undesirable effects on dyslipidemia prevalence. Therefore, the adverse side effects of these aforementioned diets should be noted by dietitians.

## Data Availability

Data will be available upon request from the corresponding author.
